# Novel Insights into the Prognosis and Immunological Value of the SLC35A (Solute Carrier 35A) Family Genes in Human Breast Cancer

**DOI:** 10.3390/biomedicines9121804

**Published:** 2021-11-30

**Authors:** Hoang Dang Khoa Ta, Do Thi Minh Xuan, Wan-Chun Tang, Gangga Anuraga, Yi-Chun Ni, Syu-Ruei Pan, Yung-Fu Wu, Fenny Fitriani, Elvira Mustikawati Putri Hermanto, Muhammad Athoillah, Vivin Andriani, Purity Sabila Ajiningrum, Chih-Yang Wang, Kuen-Haur Lee

**Affiliations:** 1 Ph.D. Program for Cancer Molecular Biology and Drug Discovery, College of Medical Science and Technology, Taipei Medical University, Academia Sinica, Taipei 11031, Taiwan; d621109004@tmu.edu.tw (H.D.K.T.); g.anuraga@unipasby.ac.id (G.A.); 2 Graduate Institute of Cancer Biology and Drug Discovery, College of Medical Science and Technology, Taipei Medical University, Taipei 11031, Taiwan; m654110001@tmu.edu.tw (D.T.M.X.); ckni1012@gmail.com (Y.-C.N.); panray8802069487@gmail.com (S.-R.P.); 3 Ph.D. Program for Cancer Molecular Biology and Drug Discovery, College of Medical Science and Technology, Taipei Medical University, Taipei 11031, Taiwan; yeas0310@hotmail.com; 4 Department of Statistics, Faculty of Science and Technology, Universitas PGRI Adi Buana, Surabaya 60234, Indonesia; fenny_f@unipasby.ac.id (F.F.); elvira@unipasby.ac.id (E.M.P.H.); athoillah@unipasby.ac.id (M.A.); 5 National Defense Medical Center, School of Medicine, Department of Medical Research, Tri-Service General Hospital, Taipei 11490, Taiwan; qrince@yahoo.com.tw; 6 Department of Biological Science, Faculty of Science and Technology, Universitas PGRI Adi Buana, Surabaya 60234, Indonesia; v.andriani@unipasby.ac.id (V.A.); puritysabila@unipasby.ac.id (P.S.A.); 7 Cancer Center, Wan Fang Hospital, Taipei Medical University, Taipei 11031, Taiwan; 8 TMU Research Center of Cancer Translational Medicine, Taipei 11031, Taiwan

**Keywords:** bioinformatics, biomarker, immunology, breast cancer, SLC35A1, SLC35A2, SLC35A3, SLC35A4, SLC35A5

## Abstract

According to statistics 2020, female breast cancer (BRCA) became the most commonly diagnosed malignancy worldwide. Prognosis of BRCA patients is still poor, especially in population with advanced or metastatic. Particular functions of each members of the solute carrier 35A (SLC35A) gene family in human BRCA are still unknown regardless of awareness that they play critical roles in tumorigenesis and progression. Using integrated bioinformatics analyses to identify therapeutic targets for specific cancers based on transcriptomics, proteomics, and high-throughput sequencing, we obtained new information and a better understanding of potential underlying molecular mechanisms. Leveraging BRCA dataset that belongs to The Cancer Genome Atlas (TCGA), which were employed to clarify SLC35A gene expression levels. Then we used a bioinformatics approach to investigate biological processes connected to SLC35A family genes in BRCA development. Beside that, the Kaplan–Meier estimator was leveraged to explore predictive values of SLC35A family genes in BCRA patients. Among individuals of this family gene, expression levels of SLC35A2 were substantially related to poor prognostic values, result from a hazard ratio of 1.3 (with 95 percent confidence interval (95% CI: 1.18–1.44), the *p* for trend (ptrend) is 3.1 × 10^−7^). Furthermore, a functional enrichment analysis showed that SLC35A2 was correlated with hypoxia-inducible factor 1A (HIF1A), heat shock protein (HSP), E2 transcription factor (E2F), DNA damage, and cell cycle-related signaling. Infiltration levels observed in specific types of immune cell, especially the cluster of differentiation found on macrophages and neutrophils, were positively linked with SLC35A2 expression in multiple BRCA subclasses (luminal A, luminal B, basal, and human epidermal growth factor receptor 2). Collectively, SLC35A2 expression was associated with a lower recurrence-free survival rate, suggesting that it could be used as a biomarker in treating BRCA.

## 1. Introduction

As specified by the most recent health statistics report from the World Health Organization (WHO), female breast cancer (BRCA) remaines one of the most frequently diagnosed cancers in women overall, with over 1 million cases recorded worldwide each year, accounting for 25% of all malignancies in women [1]. Despite the availability of advanced treatments such as chemotherapy, radiotherapy, targeted treatment, and hormonal therapy, the disease continues to claim lives, with high rates of treatment failure due to both intrinsic and acquired tumor resistance. Moreover, steady increases in BRCA incidence and mortality rates for several consecutive years have resulted in enormous health and economic burdens [2,3,4,5,6,7]. Due to the heterogeneity of this disease, at both the molecular and histological levels, the currently utilized stratification system is still undergoing adjustments. While existing evidence-based treatment strategies use traditional hormonal factors, including the positive or negative status of estrogen receptor (ER) and/or progesterone receptor (PR), and human epidermal growth factor receptor-2 (HER2) to stratify BRCA prior to determining the most suitable treatment for patients, plenty of immunohistochemical markers (IHC), such as Ki-67, p53, and E-cadherin are simultaneously employed as predictive tools for those subtypes that still lack druggable molecular targets [2,3,4,6]. BRCA is now divided into five subtypes, namely luminal A or HR-positive/HER2-negative, luminal B or HR-positive/HER2-positive, triple-negative or HR/HER2-negative (TNBC), HER2-positive, and normal-like BRCA [8]. BRCA stratification aids in selecting treatment alternatives and predicting treatment responses and additional prognoses [9,10,11].

The solute carrier (SLC) superfamily is thought to be the second-largest gene family in the human genome after well-known ATP-binding cassette (ABC) transporters, and it encodes transporters of both endogenous and exogenous substances [12]. SLCs transporter are a superfamily gene that encodes for membrane proteins. They are further categorized into 65 subfamilies based on sequence similarities and the number of anticipated or observed transmembrane α-helices [13]. They also play roles in the disposition of a variety of therapeutically important drugs, such as chemotherapeutics and antidiuretics. SLC transporters are intriguing drug targets because of their biological and pharmacological significance in addition to their functions in a variety of human illnesses [14].

Of those, Solute carrier 35 (SLC35) is a subfamily of nucleotide sugar transporters (NSTs) that transport nucleoside-sugar compounds. SLC35A1–5, SLC35B1–4, SLC35C1 and -2, SLC35D1–3, SLC35E1–4, and the subfamilies of orphan SLC35 transporters of SLC35E1–4 and SLC35F1–5 have all been documented thus far based on sequence similarities [15]. The solute carrier family 35 member A2 (SLC35A2) gene, found on the X chromosome, encodes for a multi-pass membrane protein that transports uridine diphosphate (UDP)-galactose—a glycosyl donor for synthesis of the glycan chains, from the cytosol to the lumen of Golgi apparatuses and subsequently to the endoplasmic reticulum. Galactosylation of N- and O-glycans on glycoproteins in Golgi apparatuses require the UDP-galactose transporter (UGT; SLC35A2) [16]. It is also required in the endoplasmic reticulum for synthesizing of galactosylceramide and galactosyl diglyceride [17]. Recent research discovered that mosaic variants of the SLC35A2 gene, which encodes the major Golgi-localized UGT that necessary for the glycosylation in both sphingolipid and protein, became the main cause of a rare genetic disease named congenital disorder of glycosylation (CDG) in which the majority of affected individuals suffer from varying degrees of neurological impairments, such as intellectual disabilities, dysmorphisms, and epileptic encephalopathies [18,19,20]. However, the underlying pathomechanisms remain unclear [21,22]. Aside from that, current research has found that SLC transmembrane transporters play significant roles in drug resistance to cytotoxic medicines. Since enrichment of SLC35A2- and SLC38A5-lacking cells were detected with a cisplatin treatment regimen, SLC35A2/SLC38A5 are believed to play major roles in sensitivity to cisplatin (a platinum-based chemotherapy medication used to treat several types of malignancies) [23]. Because nucleotide sugar transporters (NSTs) are primarily involved in the metabolism of glucose, which is intensely utilized by malignant tumors, a thorough understanding of this membrane protein family has become critical for cancer metabolic research [24].

Genomic network analyses using large databases, which allow us to understand activities of specific gene families, are now widely acknowledged as [25,26]. Integrative bioinformatics analyses based on transcriptomics, proteomics, and high-throughput sequencing to identify therapeutic targets for particular cancers can help us obtain new knowledge and a better understanding of probable underlying biological pathways. As stated above, although members of the SLC35A family play critical roles in carcinogenesis and tumor progression, the precise role of each member of this gene family in human BRCA is, however, almost unknown [27,28]. In this study, we attempted to conduct a complete analysis in order to improve our biological understanding of SLC35A family members based on our current knowledge, as we believe that this research will provide novel molecular therapeutic targets and their possible biological activities for BRCA patients using integrated bioinformatics. Using data from ONCOMINE, The Cancer Genome Atlas (TCGA), and other publicly available datasets, we explored SLC35A family members in BRCA. The SLC35A family’s expression profiles, clinical importance, and predictive usefulness for patients with BRCA were investigated. SLC35A2 showed biomarker potential for prognosis, and we discovered associated genes and regulatory networks, showing key molecular pathways that need to be further investigated in BRCA.

## 2. Materials and Methods

### 2.1. UALCAN

TCGA “Level 3” RNA-sequencing (RNA-Seq) together with clinical data accuired from around 30 different types of cancer are combined in UALCAN (http://ualcan.path.uab.edu, accessed on 15 July 2021). The RNA-Seq by Expectation Maximization (RSEM) tool was used to calculate expression values for 20,502 genes in this database [29]. Differences in gene expression levels across groups were measured in transcripts per million (TPM) to see if the differences were statistically significant. TCGA data were retrieved using this platform, which included 114 normal samples and 1097 primary BRCA samples. Messenger (m)RNA levels of five SLC35A family genes in BRCA, as well as their associations with clinicopathological characteristics and tumor stages, were used in our study.

### 2.2. Analyses on Data of Cancer Cell Line Encyclopedia (CCLE)

To gain better insights in molecular landscape of SLC35A family genes, genomic data, analysis, and visualization of 947 human breast cancer cell lines were acquired from The CCLE database [30]. Using a log2 transformation of expression values, expression levels of each individual were exported and shown as a heatmap.

### 2.3. Functional Enrichment Analysis

The TCGA (*n* = 1084) [14] and METABRIC datasets (*n* = 2509) [31] available for querying are from the cBioPortal platform (https://www.cbioportal.org, accessed on 27 July 2021) database [14,31,32], which were used to collect data. The MetaCore (https://portal.genego.com, accessed on 5 August 2021) was subsequently leveraged to identify more comprehensive understanding about validated biological pathways provided by experimental findings (Omics data). Beside that, DOSE R packages (version 4.0) data was utilized for Gene Ontology (GO) analysis to define genes and three functional categories, namely: biological processes (BPs), cellular components (CCs), and molecular functions (MFs), and dataset from the METABRIC database was used to conduct a gene set enrichment analysis (GSEA, http://software.broadinstitute.org/gsea, accessed on 21 August 2021) to further uncover gene product functions in female breast cancer. Finally, we calculated relevant parameters including *q*-value false discovery rate (FDR) and normalized enrichment score (NES). As previously mentioned [21,22,24,33,34,35,36], a *q*-value of <0.25 was used as the border criteria whereas a NES of >2 together with a nominal *p* value of 0.05 were considered as thresholds.

### 2.4. Survival Analysis

The product limit estimator, commonly known as the Kaplan–Meier estimator, is a non-parametric statistic that is used to estimate the survival function given lifetime data. Base on this concept, Kaplan-Meier (KM)-plot database (https://kmplot.com, accessed on 15 August 2021) were leveraged to examine whether there are correlation between SLC35A mRNA expression profiles and BRCA patient survival period. The KM-plot database was known as a robust framework that was available online for producing survival outcomes for thousands of patients with various types of cancer, primarily from TCGA datasets. Furthermore, gene expression and survival data were obtained from Affymetrix HG-U133A, HG-U133 Plus 2.0 microarrays), TCGA (HG-U133A 2.0, and the Gene Expression Omnibus (GEO). This platform, which includes microarray data from 1809 patients, contains 70,632 gene symbols related to BRCA prognoses [37]. In the KM-plot database [37], we chose recurrence-free survival (RFS) patient groups, and Jetset selects the best probe set. A hazard ratios (HRs) with 95% confidence intervals (CIs) as well as log-rank *p*-value are showed in the plot. The horizontal axis (x-axis) represents months of survival, while the vertical axis (y-axis) represents survival probabilities.

### 2.5. Overall Protein Expression Profile of SLC35A Family Genes

The Human Protein Atlas (HPA) platform, which comprises more than 10 million IHC images and 400,000 high-resolution immunofluorescences (IF) images of tissue microarrays, was used to determine SLC35A family protein expressions. These microarrays, which are tagged with over 700 antibodies against human proteins, correspond to 20 different types of human cancer [38]. We used BRCA data resources to gather 1075 patient samples and analyzed protein expressions in clinical samples through IHC images. For each cell line and antibody, the staining intensity, subcellular localization, and single-cell variability (SCV) are provided. Depending on the laser capacity and detector gain settings used for image capture, as well as the discernible appearance, the staining intensity is characterized as strong, moderate, weak, or negative. Manual staining intensity assessment of IHC data (strong, moderate, weak, or negative) and the proportion of stained cells (>75%, 25–75%, or 25%) were used to determine protein expression scores.

### 2.6. TIMER Analysis

We screened our family genes through a pool of around 10,827 tumors of 32 different cancer types using TIMER2.0 (http://timer.comp-genomics.org/, accessed on 13 September 2021) by comparing the correlation with immune infiltrations. All tumor data extracted from TCGA, with a wide range of factors such as somatic mutated cells, transcriptome profiles, and clinical outcomes, were collected from GDAC (http://firebrowse.org/, accessed on accessed on 13 September 2021). Log2[RSEM] values were used to represent gene expression levels. SLC35A genes were utilized in the DiffExp module with default settings to acquire expression levels in normal and malignant tissues. The “DiffExp” module allows researchers to compare any gene expression levels in term of normal tissues and malignant ones across all TCGA tumors. A scatterplot of gene expression levels versus tumor purity is shown, along with Spearman correlation coefficients and statistical significance, and the tumor purity can be adjusted [39].

### 2.7. Statistical Analysis

To retrieve patient data and investigate the impacts of different SLC35A family members on overall survival (OS), we accessed the TCGA Pan-Cancer Atlas, which is referred to as a dataset belong to cBioPortal (https://www.cbioportal.org, accessed on 27 July 2021). Survival study were futher carried out with the default settings. In particular, recurrence-free survival (RFS) was detailed with the best J probe set and auto-best cutoff values since the best presenting threshold was ultilized as the cutoff after considering all feasible cutoff values available in the range of lower and higher quartiles. A statistically significant log-rank *p*-value of <0.05 was used [40,41,42,43,44,45].

## 3. Results

### 3.1. Overall Analysis of Expression Profiles of SLC35A Family Members

**Figure 1:**
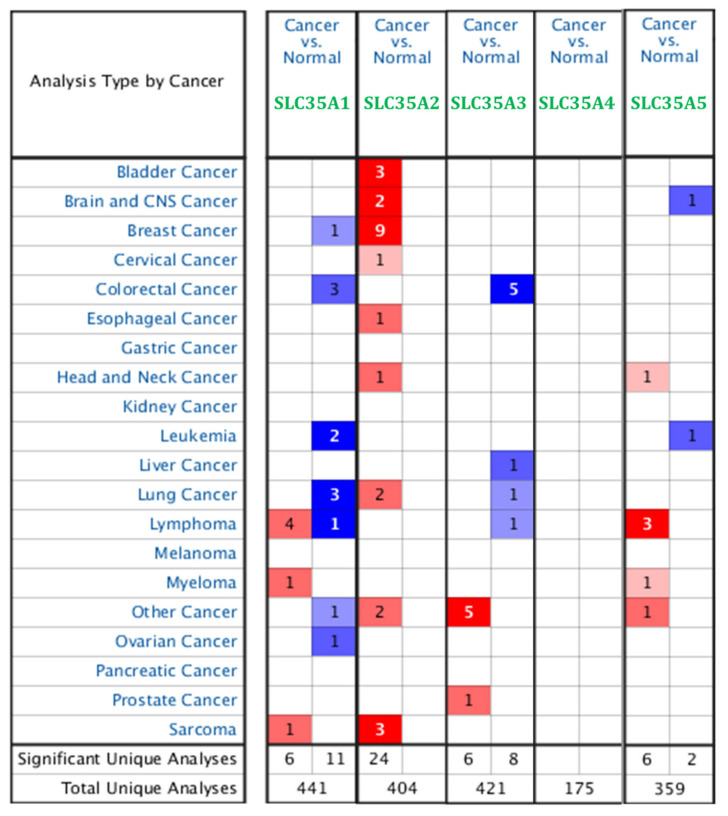
Pan-cancer analysis and overview of mRNA expression levels of solute carrier 35A (SLC35A) family genes in 20 common types and subtypes of cancer from the Oncomine platform database. Dysregulation of each individual SLC35A gene in targeted cancer tissues measured by mRNA expression levels was compared to their normal counterparts using Student’s *t*-test. The cutoff of parameters were restricted as follows: *p* < 0.05; fold change of >2; gene rank: top 10%. The number of datasets that met those thresholds is presented as a number inside the table cells while the colors indicate the trend of gene expression (red for up-regulation and blue for down-regualtion, respectively), and the intensity of the colors indicates the degree of abnormal expression.

In recent years, improvements in storing large amounts of data, particularly financial transcriptomic data, have been remarkably robust. Because there are limited data on correlations between SLC35A family genes and BRCA, we first used Oncomine to determine expression profiles of each individual of SLC35A family gene in BRCA patients. The expression of SLC35A2 mRNA was elevated in BRCA patients. SLC35A3 was also overexpressed in BRCA patients, according to one database (Figure 1). Supplementary Table S1 describes the datasets that confirmed SLC35A2 overexpression.

**Figure 2:**
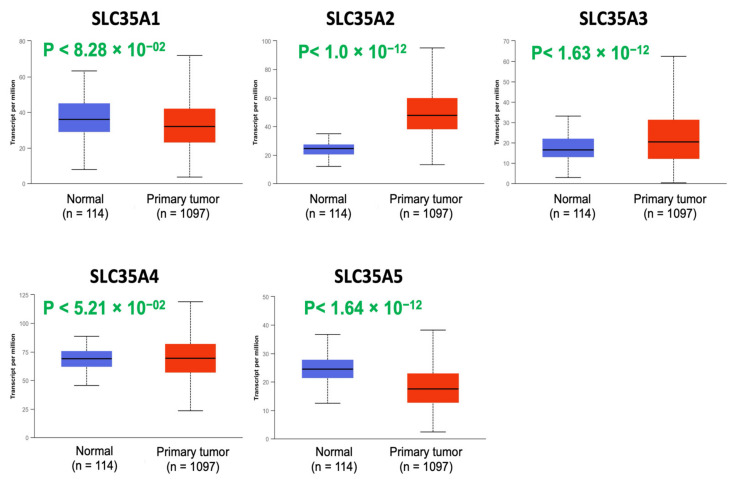
Solute carrier 35A (SLC35A) expressions in BRCA patients (1097 primary tumor samples) versus healthy counterparts (114 normal samples) extracted from UALCAN database. Boxplot of SLC35A mRNA expression levels measured in BRCA specimens (red) compared to their normal counterparts (blue). All *p* values were set to <0.001.

Expression profiles of all five members of the SLC35A family were then determined using the UALCAN database (Figure 2). mRNA levels of SLC35A2 and SLC35A3 were considerably overexpressed in BRCA tissues. SLC35A5 transcript levels were down-regulated relative to healthy controls, while SLC35A1, SLC35A4, and SLC35A3 transcript levels exhibited non-significant differences.

**Figure 3:**
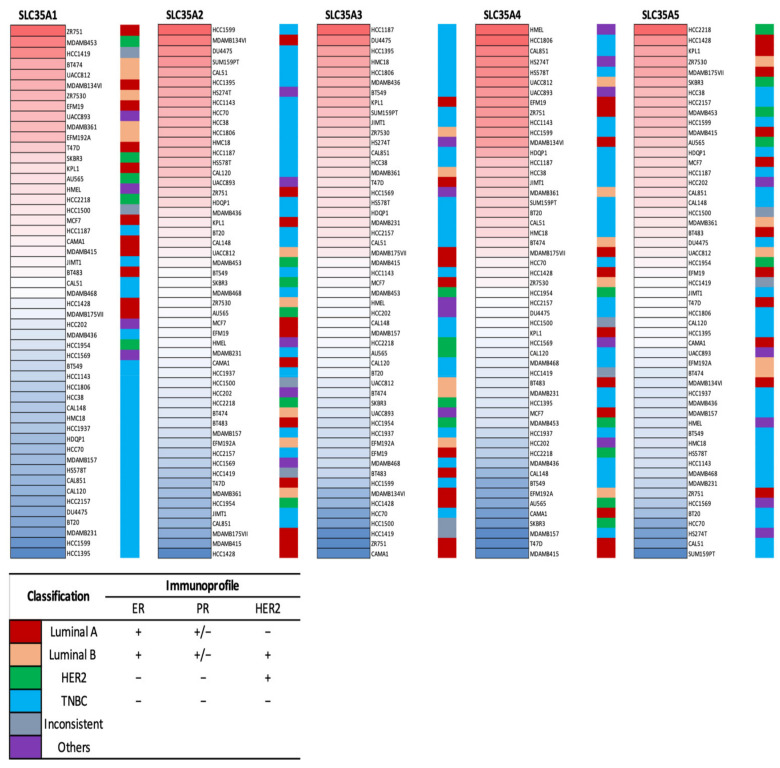
Heatmap of solute carrier 35A (SLC35A) gene mRNA expressions in multiple breast cancer cell lines (CCLE). Data table represented in different colored columns correspond to specific molecular subtype of each cell line. The term “inconsistent” refers to cell lines which were inconsistently annotated in terms of the marker status. “Others” include two non-breast cancer cell lines (HS274T, breast fibroblasts, and HMEL, engineered breast cells). TNBC stands for triple-negative breast cancer.

Expression levels of the SLC35A gene family members in multiple BRCA cell lines were further explored using the CCLE database. Triple-negative breast cancer (TNBC) cell lines like HCC1395, HCC1187, and MDAMB436 displayed high expression in SLC35A2, SLC35A3, SLC35A4, and SLC35A5, according to the CCLE analysis (Figure 3).

### 3.2. Relationships between the Expression of SLC35A Family Members and BRCA Progression

**Figure 4:**
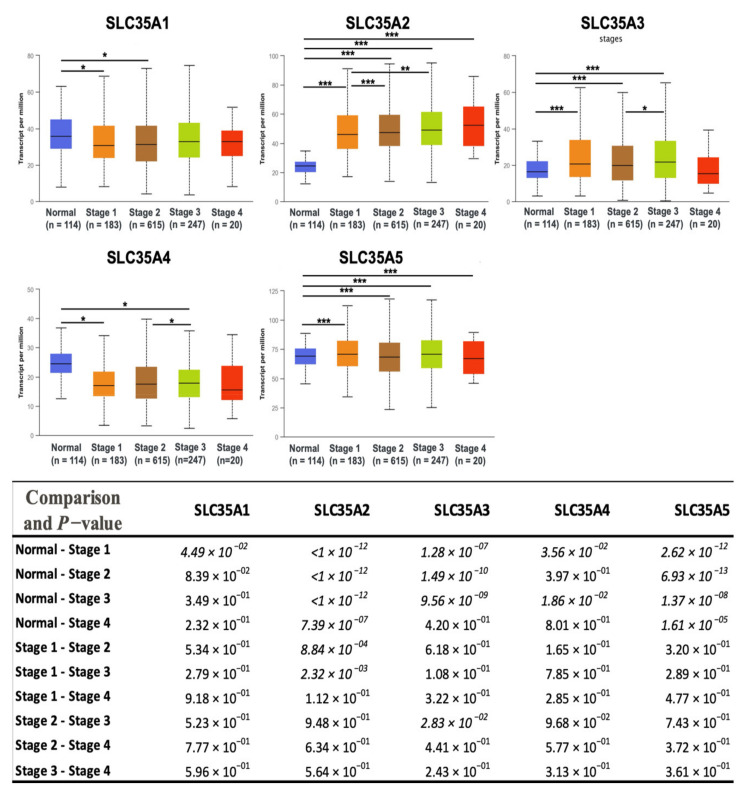
Solute carrier 35 A (SLC35A) transcription profiles in four stages of BRCA (UALCAN database). In the TCGA dataset, there were 114 normal samples, 183 patients at stage 1, 615 patients at stage 2, 247 patients at stage 3, and 20 patients at stage 4. Boxplots show relative expression levels of SLC35A family genes in healthy controls and stages 1, 2, 3, and 4 of breast cancer. Figure depicts expressions of SLC35A family genes (SLC35A1–SLC35A5) in breast cancer patients at individual cancer stages. Student’s *t*-test was conducted for statistical tests between groups. (* *p* < 0.05, ** *p* < 0.01, *** *p* < 0.001).

After determining transcriptomic levels of each SLC35A member, the UALCAN database was used to investigate the correlations between them and patients’ corresponding tumor stages. We identified a substantial link between SLC35A2 and an increase in tumor stage in BRCA patients. Correlations between the remaining SLC35A family genes and tumor stages were less obvious and statistically inconsequential (Figure 4). Supplementary Table S2 shows the total *p*-value for each stage pair.

### 3.3. SLC35A Co-Expression Analysis

**Figure 5:**
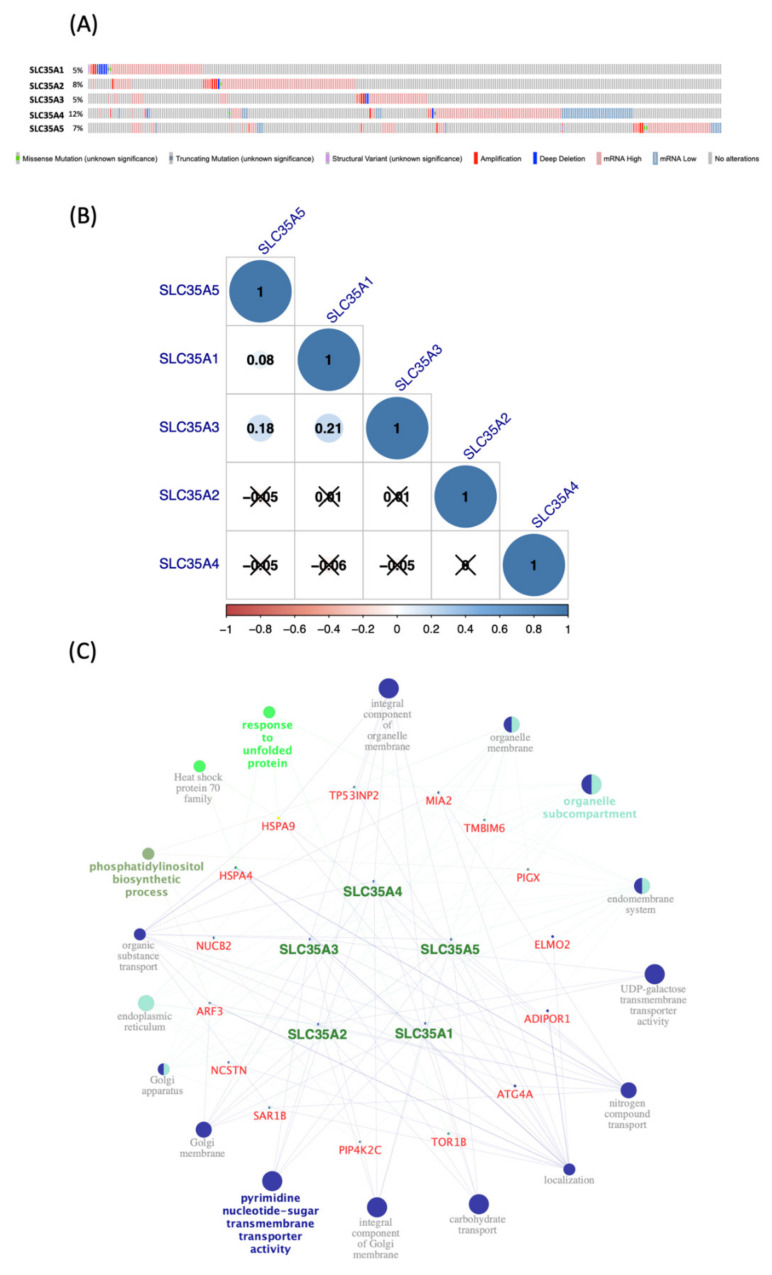
Co-expression analysis and genetic alterations in solute carrier 35A (SLC35A) family members (cBioPortal) (Cytoscape). (**A**) Alterations in SLC35As in TCGA Pan-Cancer Atlas (*n* = 1082), are summarized. Gene amplification is represented by red bars, deep deletions by blue bars, and missense mutations by green bars. This graph demonstrates that SLC35A2 (8%) and SLC35A4 (12%) have substantial rates of mutation. (**B**) Using the TCGA Pan-Cancer Atlas dataset (*n* = 1082), correlations among SLC35A family members in breast cancer were assessed. The “corrplot” R package was used to build the symmetrical correlation matrix. Color of Spearman’s rank correlation coefficients (rho) indicate the degree of pairwise correlations (rho). A positive correlation is indicated by a darker blue tone and a larger dot size, whereas a negative correlation is indicated by a darker red color and a smaller dot size. Non-significant correlation coefficient values (*p* > 0.01) are represented by a cross symbol. (**C**) A network of connected genes and pathways was created. For each SLC35A member, top 20% of co-expressed genes were shorlisted from the METABRIC database (approximately 4000 genes) then being merged prior to being intersected with a list of 21 common genes. Final result gene list was input to ClueGo of Cytosape. Only pathways which *p*-value of 0.05 are revealed since Bonferroni correction of the *p*-value were applied to account for statistical option of a two-sided hypergeometric test for enrichment.

Using the cBioPortal platform, we looked into SLC35A gene mutations in BRCA patients from 1082 patients profiled by the TCGA Pan-Cancer Atlas. Top 20% (4000 genes) of co-expressed genes list acquired from the METABRIC database were then intersected with each gene in the SLC35A family. As shown in Figure 5C, the combined result were subsequently entered into Cytoscape to build network of genes and pathways through Gene Ontology (GO) and the Kyoto Encyclopedia of Genes and Genomes (KEGG) analyses. We also investigated cross-correlations between individuals of SLC35A family gene via their mRNA expression and recorded the respective Pearson’s correlation coefficients (Figure 5B).

### 3.4. Survival Analysis of SLC35A Family Genes

**Figure 6:**
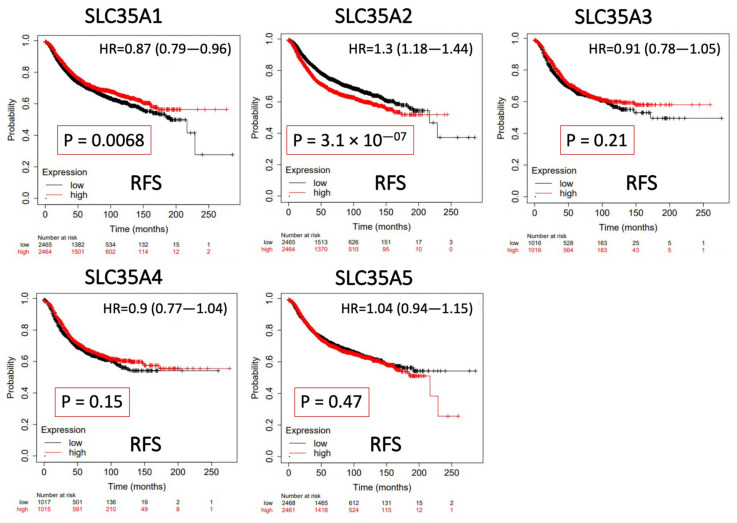
Significant correlations between mRNA levels of solute carrier 35A (SLC35A) family members and the recurrence-free survival (RFS) curve of patients diagnosed with breast cancer (BCRA). The two survival curves respectively illustrate survival outcomes (including survival percentages and survival times) of BRCA patients with high (red) or low expression (black) levels of SLC35A family members. Increased mRNA levels of SCL35A2 resulted in a poor prognosis, while increasing levels of the remaining members were associated with favorable outcomes. SLC35A3, SLC35A4, and SLC35A5 were not statistically significant (*p* < 0.05).

The predictive value of each SLC35A mRNA expression level in relation to BRCA patient survival rates was determined using a KM analysis. Only SLC35A2 was found to be substantially correlated with negative increase in prognostic outcomes of BRCA patients in terms of RFS (hazard ratio (HR) = 1.3, 95% confidence interval (CI): 1.18–1.44, *p* for trend = 3.1 × 10^−7^). Low levels of SLC35A1 expression, on the other hand, were associated with prolonged recurrence metastasis-free survival (HR = 0.87, 95% CI: 0.79–0.96, *p* for trend = 0.0068). SLC35A3, SLC35A4, and SLC35A5 were also shown to have nonsignificant predictive significance. Findings are summarized in Figure 6.

### 3.5. Protein Expressions of SLC35A Family Members

**Figure 7:**
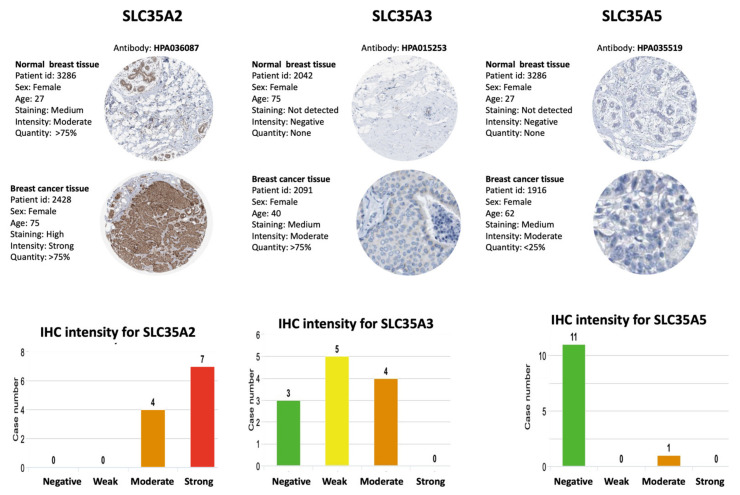
IHC expression patterns of solute carrier 35 A (SLC35A) family members in normal and BRCA tissues. IHC-stained images show the intensities of antibodies in both BRCA and adjacent normal tissues. A bar chart under each IHC stained images means the IHC staining intensity for each SLC35A members (SLC35A2, SLC35A3, SLC35A5, respectively).

In the HPA database, we looked up protein expressions of SLC35A family members. Figure 7 shows IHC images of SLC35A1, SLC35A2, SLC35A3, SLC35A4, and SLC35A5 in BRCA patients, together with clinicopathological information such as patient ID, age, and gender as well as normal and tumor samples (HPA). Overexpressed levels of the SLC35A2 and SLC35A4 proteins was discovered in tumor tissues in a way similarly to patterns reported in BRCA patient samples of the HPA dataset. These findings were consistent with mRNA expression profiles.

### 3.6. Relationship between the Transcriptomic Expression Levels of SLC35As and Various Types of Immune Cells as Biomarkers

**Figure 8:**
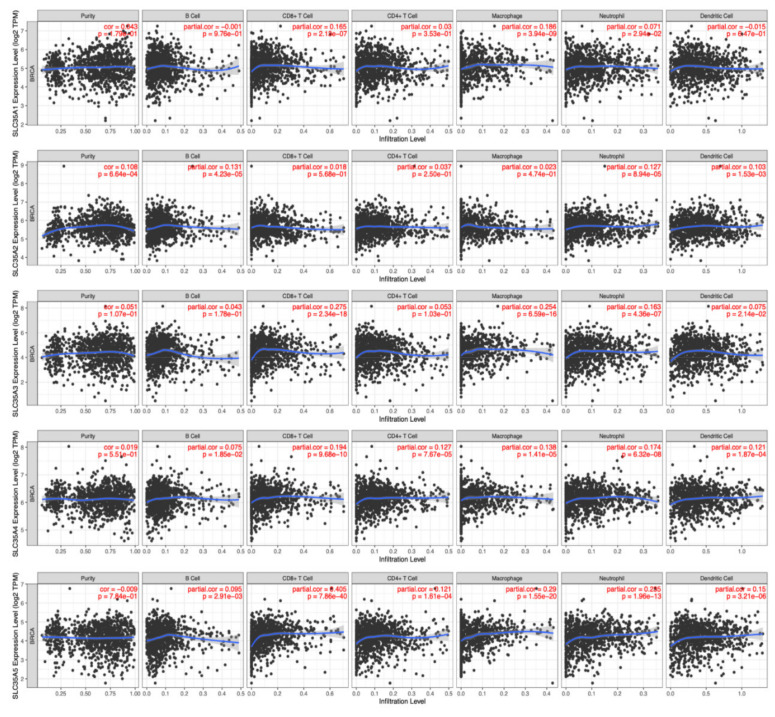
Correlations between solute carrier 35A (SLC35A) family member expressions and immune infiltration profiles of BRCA performed in the TIMER database. The graph shows above respectively depicts the correlations between abnormally expressed gene of SLC35A family and levels of some remarkable tumor-infiltrating immune cell markers, including the lymphoid lineage (B cells, CD4^+^ T cells, and CD8^+^ T cells), followed by the myeloid lineage (neutrophils, macrophages, and dendritic cells) (*p* < 0.05).

Because of their propensity to migrate from the bloodstream into solid tumors and exhibit tumoricidal effects, tumor-infiltrating lymphocytes (TILs) have gained favor as a sort of cancer immunotherapy. As a result, understanding links between SLC35A expressions and invading cells is crucial for confirming SLC35A members as BRCA immunotherapeutic targets. A tumor microenvironment (TME) that is evolving is a complicated and ever-changing entity. The TME setup varies from type to type of tumors. The TME plays a significant role in cancer progression, according to a large body of studies [46,47]. SLC35A expressions and indicators of tumor-infiltrating immune cells, such as B cells, neutrophils, M1 macrophages, M2 macrophages, DCs, and tumor-associated macrophages (TAMs), were also investigated. SLC35A1 expression showed significant positive correlations with CD8^+^ T cells (*r* = 0.165, *p* = 2.1 × 10^−7^) and macrophages (*r* = 0.186, *p* = 3.94 × 10^−9^). SLC35A2 was significantly associated with B cells (*r* = 0.121, *p* = 4.23 × 10^−5^), neutrophils (*r* = 0.127, *p* = 8.94 × 10^−5^) and DCs (*r* = 0.103, *p* = 1.53 × 10^−3^). Similarly, SLC35A3 expression was correlated with CD8^+^ T cells (*r* = 0.275, *p* = 2.34 × 10^−18^), macrophages (*r* = 0.254, *p* = 6.59 × 10^−16^), and neutrophils (*r* = −0.163, *p* = 4.36 × 10^−7^). SLC35A4 was associated with CD8^+^ T cells (*r* = 0.194, *p* = 9.68 × 10^−10^), CD4^+^ T cells (*r* = 0.127, *p* = 7.67 × 10^−5^), macrophages (*r* = 0.138, *p* = 1.4 × 10^−5^), and neutrophils (*r* = 0.174, *p* = 6.32 × 10^−8^). SLC35A5 was correlated with CD8^+^ T cells (*r* = 0.405, *p* = 7.86 × 10^−40^), CD4^+^ T cells (*r* = 0.121, *p* = 1.61 × 10^−4^), macrophages (*r* = 0.29, *p* = 1.55 × 10^−20^), neutrophils (*r* = 0.285, *p* = 1.96 × 10^−13^), and DCs (*r* = 0.15, *p* = 3.21 × 10^−6^) (Figure 8).

**Figure 9:**
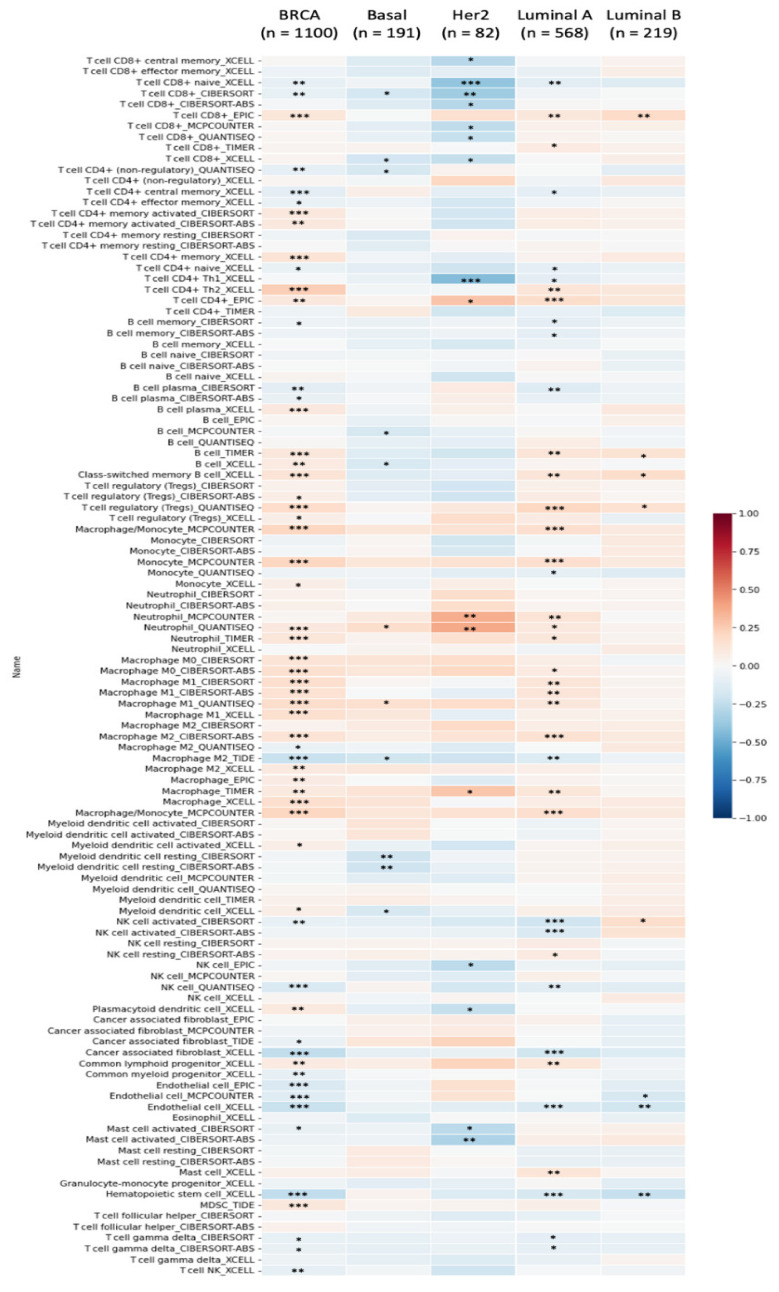
Solute carrier 35A (SLC35A2) expressions and immune infiltration in several BRCA subtypes are depicted in a heatmap. The plot shows correlations between BRCA subtypes (luminal A, luminal B, HER2, and basal) while six cutting-edge algorithms (including quanTIseq, CIBERSORT, MCP-counter, EPIC, xCell, and TIMER) were used to determine the number of samples from 112 immunological infiltrates. The R-scores varied from −1.0 to 1.0. While r = 1 represents perfect positive correlation, a perfect negative correlation is represented by r = −1 (* *p* < 0.05, ** *p* < 0.01, *** *p* < 0.001).

Furthermore, a strong connection between SLC35A2 expression in BRCA and stained samples using the HPA platform was observed, while connections of the remaining genes were less obvious, meaning that, except for SLC35A2, those aforementioned genes may not show considerably potential for being prognostic biomarkers of BRCA. In addition, while looking into the detailed analysis obtained from TIMER database, we noticed that it was high SLC35A2 expressions that presents in cancer cells and in the vast majority of immune cells that invaded BRCA tumors and subtypes. We further investigated connections between SLC35A2 expression and a wide spectrum of immune cells using the TIMER platform’s quantification methods (xCell, CIBERSORT, EPIC, quanTIseq, MCP-counter, and TIMER). SLC35A2 had the largest positive connections with CD4^+^ T cells, macrophages, and neutrophils, as illustrated in Figure 9. In contrast, SLC35A2 was found to have negative correlations with cancer-associated fibroblast XCELLs, and CD8^+^ T cells in the HER2 subtype; natural killer (NK) cell-activated CIBERSORT-ABS in the luminal A subtype, and hematopoietic stem cells in the luminal B subtype. Therefore, we further explored SLC35A2 by investigating BRCA subtype survival outcomes and GO analysis was subsequently used to determine the biological roles and activities of its gene products and complexes. The patients belong to high-expression group of SLC35A2 performed a decreased rate of OS compared to the low-expression group (HR = 1.49, 95% CI: 1.26–1.75, *p* for trend = 1.4 × 10^−6^). Similarly, in the ER^+^ (HR = 1.57, CI: 1.2–2.06, *p* = 0.001) and HER2^+^ subtypes (HR = 1.68, CI: 1.18–2.39, *p* = 0.0039), survival curves revealed shorter survival periods for patients with higher expression of SLC35A2 (Supplementary Figure S2).

### 3.7. Comprehensive Results of SLC35A2 in a Functional Enrichment Analysis

#### 3.7.1. GO Enrichment Analysis

**Figure 10:**
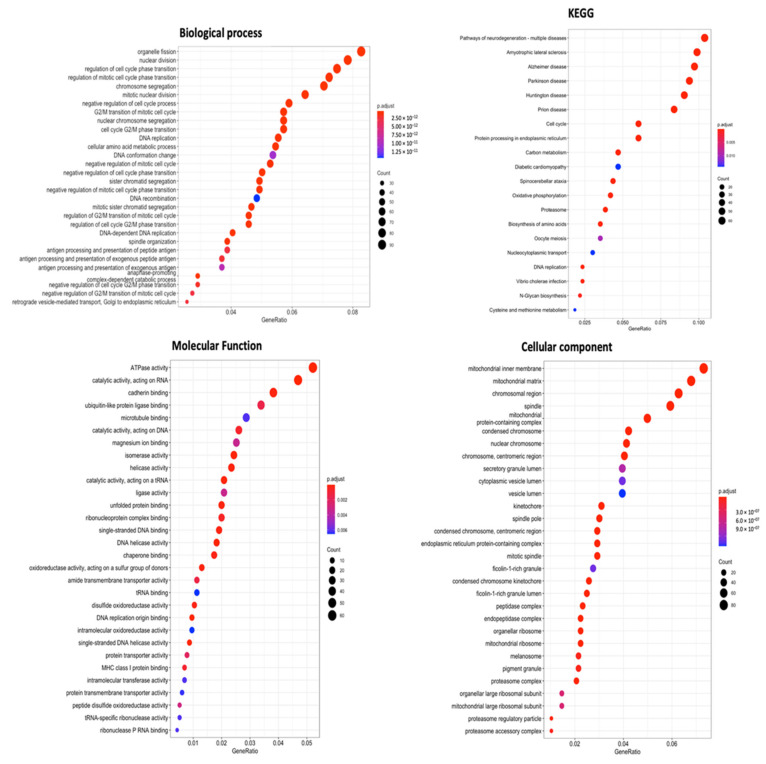
Gene ontology (GO) keywords with gene *p* values (cellular components, biological processes, molecular functions, and KEGG ontology). Number of genes counted in each function is represented by the size of the circles, and the colors of the bubbles correlate with *p* values.

For a more thorough investigation, combined data from METABRIC and TCGA Pan-Cancer databases were prepared for GO enrichment analysis. As observation from final results, particularly CCs, BPs, MFs, and KEGG ontology analysis, SLC35A2 was found to be linked to organelle fission in BPs. On the other hand, the CC analysis also revealed specific localization in mitochondrial structures: inner membrane, mitochondrial matrix, and mitochondrial complex, respectively. Lastly, high SLC35A2 expression in BRCA tumors was strongly associated with “catalytic activity, acting on RNA” and “ATPase activity,” according to MF results, while KEGG ontology revealed its role in multiple neurodegenerative diseases and additional illness-related pathways, namely, Alzheimer’s disease, prion disease, and amyotrophic lateral sclerosis (Figure 10).

#### 3.7.2. High Expression of SLC35A2 Is Related to to the E2 Transcription Factor (E2F) Target and Pro-Cancerous Related Gene Sets in BRCA

**Figure 11:**
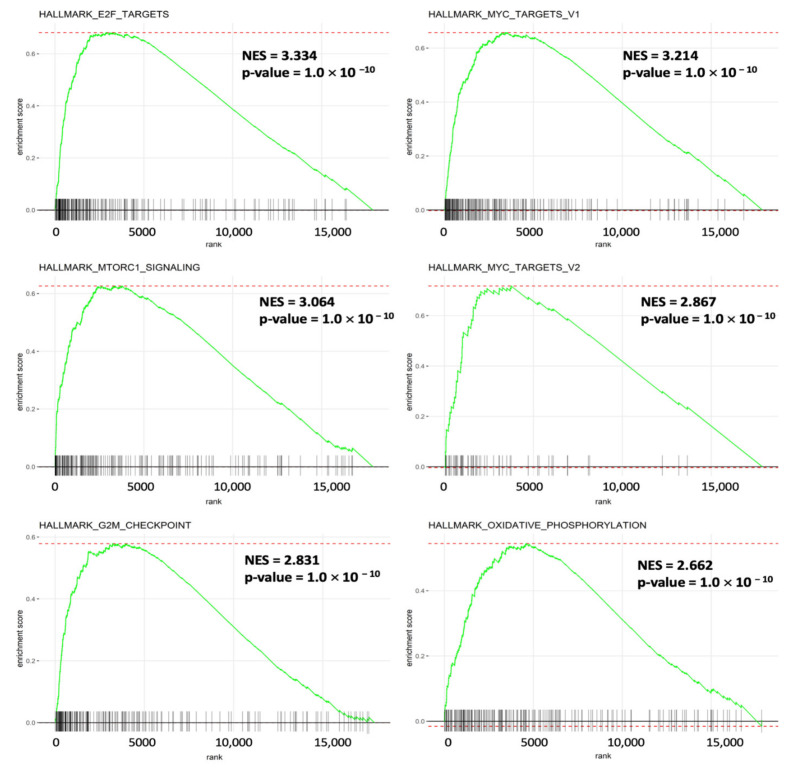
Results of a gene set enrichment analysis (GSEA) in breast cancer patients in TCGA cohort with high solute carrier 35A2 (SLC35A2) expression. Patients were divided into two halves depending on their SLC35A2 mRNA expression levels in the TCGA Pan-Cancer dataset; a corresponding ranking list of genes was then obtained and entered into the GSEA. Statistical significance was determined using a false detection rate (FDR) value of 0.25, a normalized enrichment score |NES| of >2, and a nominal *p*-value of 0.05, as recommended by the GSEA database. Enrichment at the top of the list is represented by a positive NES value, which depicts the enrichment pathway in the list.

It was fascinating to learn more about the molecular mechanisms driving gene sets that are co-expressed with SLC35A2. Enrichment of MSigDB Hallmark gene sets in BRCA samples with high SLC35A2 expression levels was investigated using a GSEA. Interestingly, E2F—targeted pathways, a factor that plays essential roles for cell proliferation control, had the highest NES (of 3.34). Recent research revealed additional roles for this pathway, particularly E2F transcription factors, in tumor progression, angiogenesis, and metastasis. Independent of clinical parameters, specific E2Fs have prognostic value in BRCA. We also discovered that inflammation and immune-related gene sets of MYC target V1, MTORC1 signaling, and MYC target V2 were enriched with high SLC35A2 expression in BRCA. Furthermore, SLC35A2 was shown to be highly expressed in a group of cancer-related genes, including the G2M checkpoint and oxidative phosphorylation (Figure 11). Supplementary Figure S1 shows the detailed enrichment results.

#### 3.7.3. SLC35A2 Plays an Important Role in Cell-Cycle Regulation

**Figure 12:**
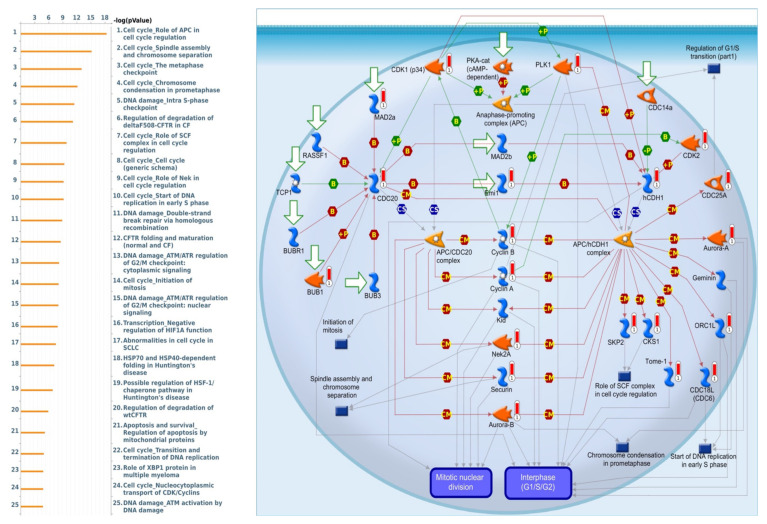
List of co-expressed genes with Solute carrier 35A2 (SLC35A2) co-expressed in BRCA database was analyzed using a MetaCore enrichment pathway analysis. The strategy was to compile lists of the top 10% expressed genes from both the METABRIC (2000 genes) and Pan-Cancer (1800 genes) prior to overlapping them to create a total gene list of 1201. The gene list was taken from TCGA Pan-Cancer Atlas and METABRIC BRCA patient databases while the pathway list was organized by the −log[*p*-value]. When doing the biological process analysis, the route “Cell cycle_Involvement of APC in cell cycle regulation” was at the top of the list.

MetaCore is frequently used to investigate network pathways based on an input gene list in order to trigger BPs. Since the gene list obtained from the intersection of the TCGA and METABRIC datasets was used as input for the following MetaCore analysis, we identified some intriguing results relating to SLC35A2. It was particularly linked to a variety of cell-cycle signaling pathways, such as “Cell cycle Role of APC in cell cycle regulation”, “Cell cycle_Spindle assembly and chromosome separation”, “Cell cycle_ The metaphase checkpoint”, and “Cell cycle Chromosome condensation in prometaphase”. Meanwhile, SLC35A2 was also correlated with “DNA damage Double-strand break repair via homologous recombination”, and “Transcription-Negative regulation of HIF1A function”, which are considered as BRCA-related immunological and cell-cycle pathways that are crucial to tumor development. Details of aforementioned pathway list and networks are depicted in Figure 12.

## 4. Discussion

The SLC35A subfamily has been widely explored to date. Three of the five proteins in this subfamily have been assigned substrate specificity. SLC35A1 is believed to be involved in the delivery of cytidine 5’-monophosphate (CMP)-sialic acid, SLC35A2 for UDP-galactose transport, and SLC35A3 for the uptake of UDP-N-acetylglucosamine. The roles of SLC35A4 and SLC35A5 in glycosylation, on the other hand, have yet to be determined. To the best of our knowledge, since previous studies did not identify the roles of *SLC35A* family genes in cancer, this is first and foremost a study that assess the potential therapeutic and prognostic values of all individuals belonged to the *SLC35A* gene family, prior to specifically examining the roles of each member in BRCA.

SLC35A1 transports CMP-sialic acid, a nucleotide sugar, into the lumen of the Golgi apparatus and glycosylates it there [48]. A previous study revealed that the glycosylation/sialylation of cell surface receptors has significant impacts on intracellular signaling pathways which can modify the biological properties of cancer cells [49,50,51,52]. For instance, the α2,6-sialylation epidermal growth factor receptor (EGFR) modulate activation of EGFR signaling whereas the loss of sialylated N-glycans on colony-stimulating factor 3 receptor (CSF3R) resulted in ligand-independent receptor activation and oncogenesis [51,52,53,54]. Furthermore, genetic screening using the cytolytic vesicular stomatitis virus (VSV) also found that deletion of the SLC35A1/CMP-sialic transporter was involved in the apoptotic response generated by VSV, providing new information about the cellular response to oncolytic viral infections [55]. Consequently, in our study, the Oncomine analysis among 20 types of cancer indicated that SLC35A1 was differentially expressed in a remarkable number of cases and was found to be down-regulated in BRCA. Inconsistent with the Oncomine analysis, reduced transcription levels of SLC35A1 have been linked to to poor distant metastasis-free survival outcomes.

In mammals, SLC35A3 is believed to be the primary UDP-N-acetylglucosamine transporter (NGT) [56]. A point mutation in the SLC35A3 gene was recently shown to produce complicated vertebral deformities in animals due to defective UDP-GlcNAc transport into Golgi vesicles, while high SLC35A3 expression was discovered to be a predictive diagnostic for patients with pancreatic ductal cancer (PDAC) [57]. The Oncomine analysis show no relationship between SLC35A3 transcription and BRCA; however, analyses of IHCstaining images together with evaluation of tumor-infiltrating immune cells suggested that SLC35A overexpression is positively correlated with CD8^+^ T-cell and macrophage infiltration. Since recent studies affirmed that the presence of CD8^+^ T-cells in BRCA helps enhance the antitumor immunity thereby resulting in better clinical outcomes [58,59,60], our study suggests that the simultaneous presence of SLC35A3 and CD8^+^ T-cells in BRCA could help better stratify patients who are certainly expected to benefit from immunotherapy regiments.

SLC35A4 was described as a possible UDP-sugar (perhaps Gal) transporter that is mostly found in the Golgi apparatus [61]; however, current research suggests that its role may be modulatory or regulatory in relation to the UGT (SLC35A2) and NGT (SLC35A3), particularly in relocation of the SLC35A2/SLC35A3 complex [62]. SLC35A5 is the only member of the SLC35 family with putative UDP-sugar-binding sites among numerous di-acidic motifs disclosed in the cytosolically situated C-terminal tail [63]. In addition to being the first mammalian NST found that transports three separate UDP-sugars (UDP-GlcA, UDP-GalNAc, and UDPGlcNAc), the SLC35A5 protein may also be a superior regulatory protein that regulates the actions of all other SLC35A subfamily members. On the other hand, according to Njiaju et al., *SLC35A5*, along with *SLC31A2* and *SLC41A2*, may play roles in drug efflux and paclitaxel cellular susceptibility [64]. Lower expressions of the aforementioned genes were likewise linked to increased paclitaxel sensitivity, intracellular drug accumulation, apoptosis, and cytotoxicity in that investigation. Both Oncomine and subsequent analyses in our study showed no clear relationships of SLC35A4, or SLC35A5, with survival times or immune infiltration levels in BRCA.

Yates et al. reviewed SLC35A2, which codes for the only known transporter of UDP-galactose to the Golgi apparatus and was previously known to be positioned on the X chromosome [65]. Congenital disorders of glycosylation (CDGs) are inherited condition that causes neurological issues and other abnormalities such as intellectual incapacity, dysmorphisms, and epileptic encephalopathies. They are caused by a mutation at Gly266Val [66]. According to Mohammad et al., lactose synthesis is slowed by genes governing UDP-galactose trafficking into the Golgi apparatus, while signal transducer and activator of transcription 5 (STAT5) is downstream of the prolactin receptor. This may, in turn, drive SLC35A2 expression, and progesterone withdrawal could be the signal that triggers a significant rise in prolactin receptor expression and so prolactin signaling [67]. In addition, we discovered that high levels of SLC35A2 expression were associated with poor prognostic characteristics and shorter survival period in women diagnosed with hormone receptor-positive BRCA (ER^+^, PR^+^, and HER^+^), implying that there is a link among lactation, sex hormone secretion, and hormone receptor-positive BRCA.

Interestingly, T cells release cytokines that are crucial to the immune system’s effectiveness. Most scientists believe that solid tumors are linked to a pathologic shift toward T-helper type 2 (Th2) cytokine release that T-helper 1 (Th1)-induced inflammation suppresses tumor growth [68,69]. Consistently, our findings also indicated the same result of high SLC35A2 expression being positively correlated with regulatory T cells (Tregs). Interestingly, one of most recent approved antibody-based immunotherapy by the US Food and Drug Administration (FDA) was anti-programmed death (PD)-1 that blocks checkpoints on T cells. Furthermore, the HER^+^ subtype is strongly linked with immune effector monocytes and tumoricidal neutrophils, which are known to be inhibitors of BRCA tumor growth [33]. Choi et al. [70] emphasized the key role of tumor-associated macrophages (TAMs) in BRCA, which also supports our results, which indicated that immune infiltration of M0 macrophages, M1 macrophages, and even M2 macrophages is highly correlated with SLC35A2 upregulation, and these TAMs have emerged as key players in tumor progression, with the potential as future treatment [71]. In addition, a gene set enrichment analysis (GSEA) indicated that SLC35A2 is a key E2F target, a transcription factor involved in several types of cancer, including pancreatic adenocarcinoma [72] and hepatocellular carcinoma [73], as well as expressing the immune-related impact on ER^+^/HER2^−^ BRCA [74]. By extracting the co-expression gene list, we also found that the mammalian target of the rapamycin C1 (mTORC1) signaling pathway was associated with SLC35A2 expression. In previous studies, this pathway was determined to contribute to the pathogenesis of psoriasis and regulate proinflammatory macrophage function and metabolism. Kim et al. reviewed the role of the mTORC1 signaling pathway in cancer development and the TME, therefore providing pieces of evidence to enhance our findings [75]. Collectively, SLC35A2 could serve as a potential marker not only in prognostics but also in immunotherapies.

Finally, our findings revealed that SLC35A2 expression of the UDP-galactose transporter (UGT) is enhanced in BRCA tissues and cell lines, with varying expression levels in distinct subtypes. In hormone receptor-positive subtypes of breast cancer (viz., ER^+^, PR^+^, and HER^+^), the SLC35A2 expression level was likewise associated with poorer prognostic features and poor survival. Furthermore, IHC revealed that SLC35A2 protein levels were significantly higher in BRCA tissues than in normal ones, and the TIMER analysis revealed a correlation between SLC35A2 and immune infiltration patterns, suggesting that SLC35A2 may play a role in BRCA diagnosis and inflammation.

The primary limitation of this work is the retrospective nature of the transcriptome analysis, that may require additional validation in larger scale studies to evaluate the feasibility of SLC35A2 expression as novel biomarker in diagnosis and treament of female breast cancer. Using high-throughput technologies, which are often used as systemic approaches, we evaluated changes in expressions of hundreds of genes for biological and genomic systems. As a result, proper and comprehensive use of multi-omics data will speed up the identification of critical disease biomarkers and assist the construction of improved molecular signatures.

## 5. Conclusions

Our findings demonstrated that SLC35A2 overexpression should be further evaluated as a indicator for poor prognosis and possible biomarker for breast cancer stratification. Nonetheless, the primary limitation of this study, that may exist due to the retrospective nature of transcriptome profilling analysis, could be addressed in future research by necessary validation through further in-depth experiments.

## Data Availability

CBioPortal: https://cbioportal.org, accessed on 27 July 2021; Human Protein Atlas: https://www.proteinatlas.org, accessed on 27 July 2021; Kaplan–Meier-plot database https://kmplot.com, accessed on 15 August 2021, MetaCore Analysis https://portal.genego.com, accessed on 13 September 2021. The datasets used and/or analyzed during the current study are available from the corresponding author on reasonable request.

## Supplementary Materials

The following are available online at https://www.mdpi.com/article/10.3390/biomedicines9121804/s1, Figure S1: SLC35A2 gene set enrichment analysis, Figure S2: Recurrence metastasis-free survival (RFS) analysis of SLC35A2 in breast cancer (Kaplan–Meier plot), Figure S3: Overall survival analysis of SLC35A family genes across several types of cancer (Kaplan–Meier plot), Figure S4: Solute carrier 35A2 (SLC35A2) co-expressed gene list in a breast cancer database was analyzed using a MetaCore en-richment pathway analysis. Table S1: Basic characteristic of SLC35A2 gene on Oncomine database, Table S2: stastistical values of SLC35A family genes expression base on individual cancer stages, Table S3: Pathway analysis of SLC35A2-coexpressed genes from public breast cancer databases using the MetaCore database.
