# Supplementary material for: Novel Insights into the Prognosis and Immunological Value of the SLC35A (Solute Carrier 35A) Family Genes in Human Breast Cancer
Source: Biomedicines. 2021 Nov 30;9(12):1804. doi: 10.3390/biomedicines9121804 (PMC8698499; doi:10.3390/biomedicines9121804)
Supplement: Supplementary file 1 [file biomedicines-09-01804-s001.zip › biomedicines-1446481-supplementary.pdf]

Supplementary data

Table S1. Basic characteristics of the solute carrier 36A2 (SLC35A2) gene in the Oncomine database

Gene	*Dataset*	*group comparison*	*fold change*	*p-value*	*number of patients*
**SLC35A2**	Curtis Breast (n=2136)	Invasive Breast Carcinoma vs. Normal	2,056	6.86E-09	21/165
		Mucinous Breast Carcinoma vs. Normal	2,136	3.6E-26	46/190
		Invasive Ductal Breast Carcinoma vs. Normal	2,295	7.3E-88	1556/1700
		Ductal Breast Carcinoma in Situ vs. Normal	2,195	1.14E-04	10/154
		Medullary Breast Carcinoma vs. Normal	2,397	1.73E-13	32/176
	TCGA breast (n=593)	Invasive Lobular Breast Carcinoma vs. Normal	2,212	1.63E-18	36/97
		Invasive Breast Carcinoma vs. Normal	2,462	5.88E-31	76/137
		Mix Lobular and Ductal Breast carcinoma vs. Normal	2.151	1.52E-6	7/68
		Invasive Ductal Breast Carcinoma vs. Normal	2,141	1,02E-38	389/450
		Male Breast Carcinoma vs. Normal	2.713	3.13E-7	3/64
		Intraductal Cribriform Breast Adenocarcinoma vs. Normal	2,539	4.52E-4	3/64
	Ma breast 4 (n=66)	Invasive Ductal Breast Carcinoma Epithelia vs. Normal	2,259	1.31E-04	9/23
		Ductal Breast Carcinoma in Situ Epithelia vs. Normal	2,234	1.23E-4	9/23

Table S2. Statistical values of solute carrier 35A (SLC35A) family gene expressions based on individual cancer stages

**Comparison**	**P-value**				
	**SLC35A1**	**SLC35A2**	**SLC35A3**	**SLC35A4**	**SLC35A5**
**Normal - Stage 1**	*4,49E-02*	*<1E-12*	*1,28E-07*	*3,56E-02*	*2,62E-12*
**Normal - Stage 2**	8,39E-02	*<1E-12*	*1,49E-10*	3,97E-01	*6,93E-13*
**Normal - Stage 3**	3,49E-01	*<1E-12*	*9,56E-09*	*1,86E-02*	*1,37E-08*
**Normal - Stage 4**	2,32E-01	*7,39E-07*	4,20E-01	8,01E-01	*1,61E-05*
**Stage 1 - Stage 2**	5,34E-01	*8,84E-04*	6,18E-01	1,65E-01	3,20E-01
**Stage 1 - Stage 3**	2,79E-01	*2,32E-03*	1,08E-01	7,85E-01	2,89E-01
**Stage 1 - Stage 4**	9,18E-01	1,12E-01	3,22E-01	2,85E-01	4,77E-01
**Stage 2 - Stage 3**	5,23E-01	9,48E-01	*2,83E-02*	9,68E-02	7,43E-01
**Stage 2 - Stage 4**	7,77E-01	6,34E-01	4,41E-01	5,77E-01	3,72E-01
**Stage 3 - Stage 4**	5,96E-01	5,64E-01	2,43E-01	3,13E-01	3,61E-01

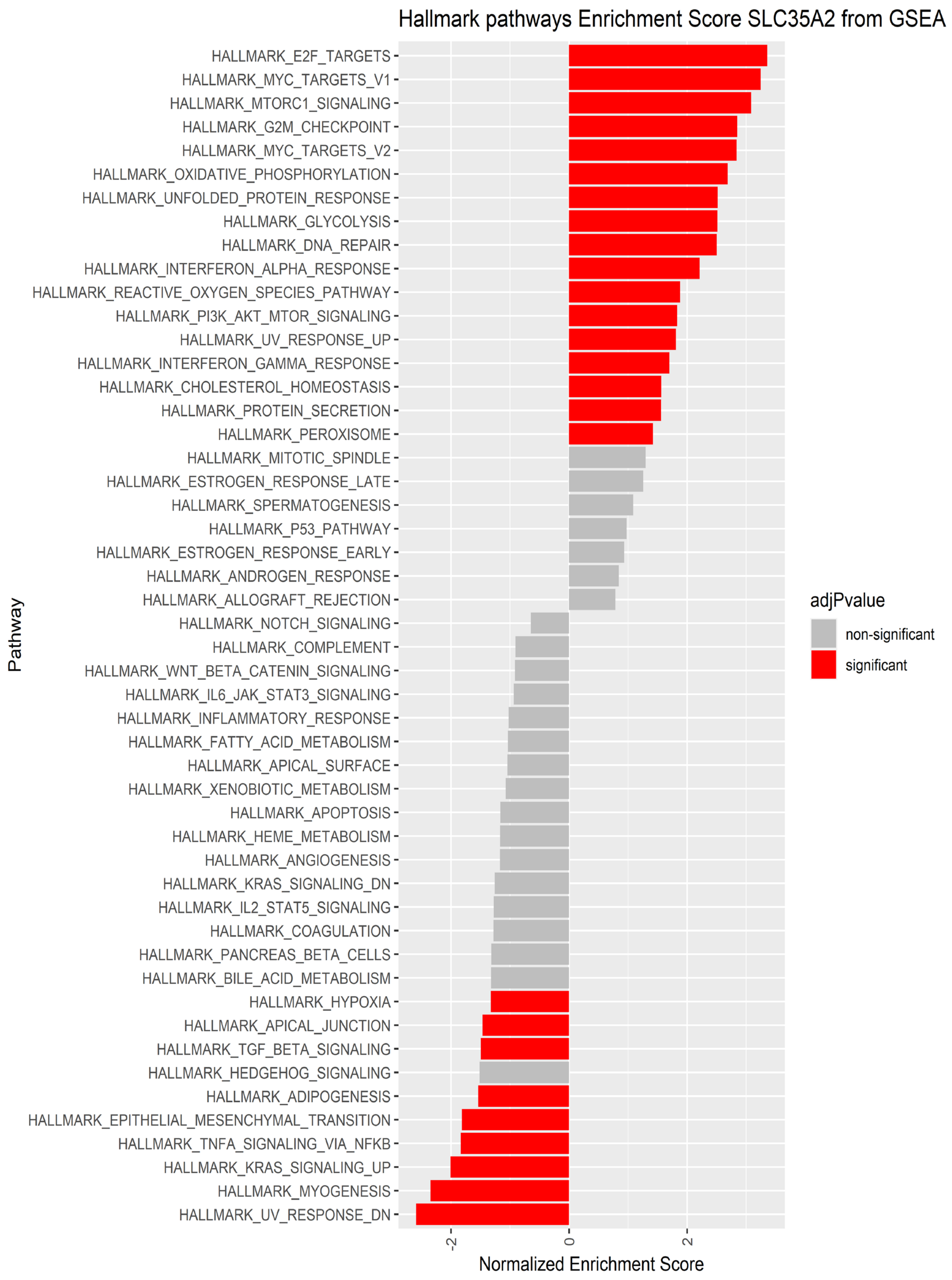

Hallmark pathways Enrichment Score SLC35A2 from GSEA
Pathway
HALLMARK_E2F_TARGETS
HALLMARK_MYC_TARGETS_V1
HALLMARK_MTORC1_SIGNALING
HALLMARK_G2M_CHECKPOINT
HALLMARK_MYC_TARGETS_V2
HALLMARK_OXIDATIVE_PHOSPHORYLATION
HALLMARK_UNFOLDED_PROTEIN_RESPONSE
HALLMARK_GLYCOLYSIS
HALLMARK_DNA_REPAIR
HALLMARK_INTERFERON_ALPHA_RESPONSE
HALLMARK_REACTIVE_OXYGEN_SPECIES_PATHWAY
HALLMARK_PI3K_AKT_MTOR_SIGNALING
HALLMARK_UV_RESPONSE_UP
HALLMARK_INTERFERON_GAMMA_RESPONSE
HALLMARK_CHOLESTEROL_HOMEOSTASIS
HALLMARK_PROTEIN_SECRETION
HALLMARK_PEROXISOME
HALLMARK_MITOTIC_SPINDLE
HALLMARK_ESTROGEN_RESPONSE_LATE
HALLMARK_SPERMATOGENESIS
HALLMARK_P53_PATHWAY
HALLMARK_ESTROGEN_RESPONSE_EARLY
HALLMARK_ANDROGEN_RESPONSE
HALLMARK_ALLOGRAFT_REJECTION
HALLMARK_NOTCH_SIGNALING
HALLMARK_COMPLEMENT
HALLMARK_WNT_BETA_CATENIN_SIGNALING
HALLMARK_IL6_JAK_STAT3_SIGNALING
HALLMARK_INFLAMMATORY_RESPONSE
HALLMARK_FATTY_ACID_METABOLISM
HALLMARK_APICAL_SURFACE
HALLMARK_XENOBIOTIC_METABOLISM
HALLMARK_APOPTOSIS
HALLMARK_HEME_METABOLISM
HALLMARK_ANGIOGENESIS
HALLMARK_KRAS_SIGNALING_DN
HALLMARK_IL2_STAT5_SIGNALING
HALLMARK_COAGULATION
HALLMARK_PANCREAS_BETA_CELLS
HALLMARK_BILE_ACID_METABOLISM
HALLMARK_HYPOXIA
HALLMARK_APICAL_JUNCTION
HALLMARK_TGF_BETA_SIGNALING
HALLMARK_HEDGEHOG_SIGNALING
HALLMARK_ADIPOGENESIS
HALLMARK_EPITHELIAL_MESENCHYMAL_TRANSITION
HALLMARK_TNFA_SIGNALING_VIA_NFKB
HALLMARK_KRAS_SIGNALING_UP
HALLMARK_MYOGENESIS
HALLMARK_UV_RESPONSE_DN
-2
0
2
Normalized Enrichment Score
adjPvalue
non-significant
significant

Figure S1. Solute carrier 35A2 (SLC35A2) gene set enrichment analysis. Bar chart shows the hallmark pathways scores related to SLC35A2. Red bars mean significant adjust-ed p values, while gray bars indicate insignificant values. A normalized enrich-ment score ǀNESǀ of >2 was set as the threshold.

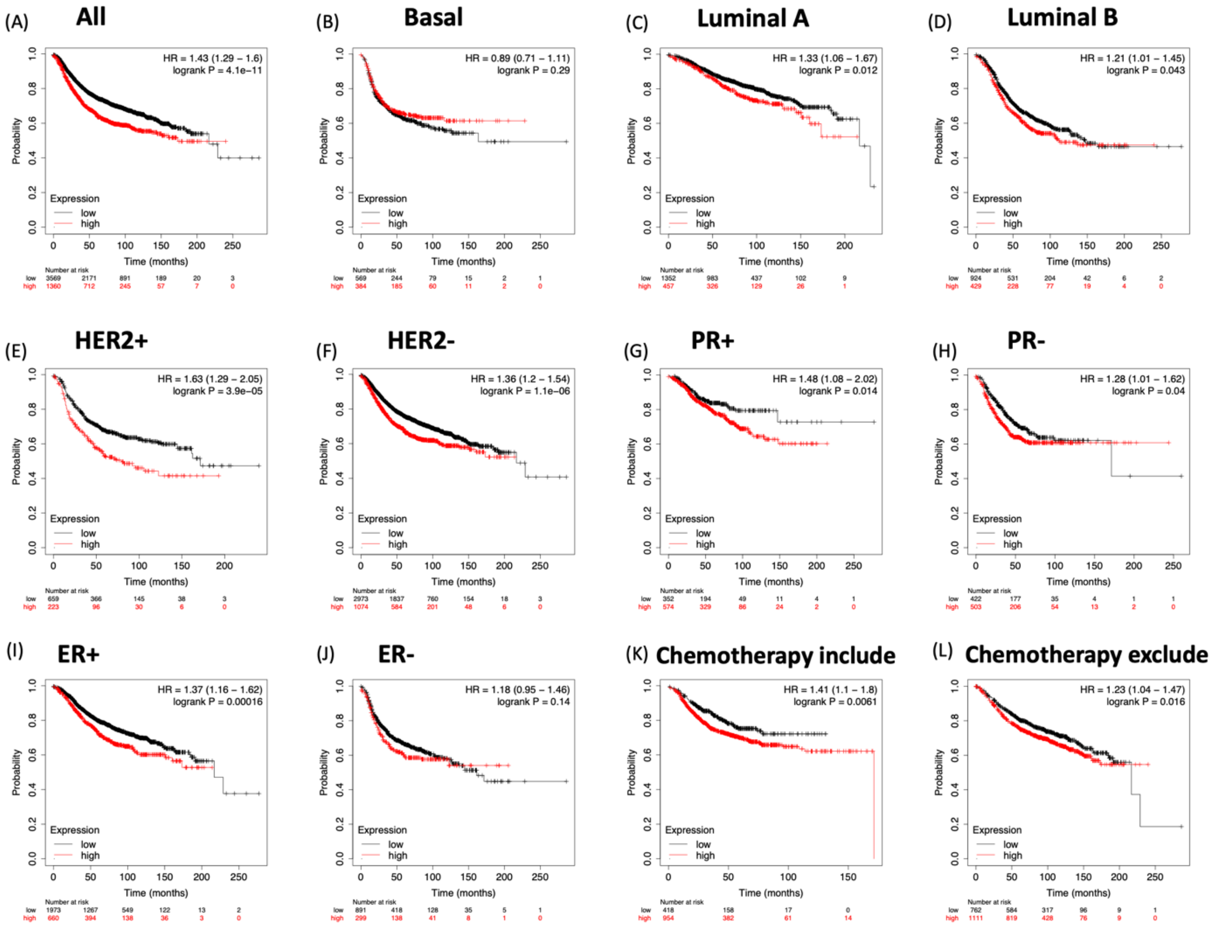

Figure S2. Recurrence metastasis-free survival (RFS) analysis of solute carrier 35A2 (SLC35A2) in breast cancer (Kaplan-Meier plot). Red and black curves represent survival analysis for higher and lower SLC35A mRNA expression levels, respec-tively. ER, estrogen receptor; HER2, human epidermal growth factor receptor 2; PR, progesterone receptor; +, positive; -, negative; chemotherapy included, excluded.

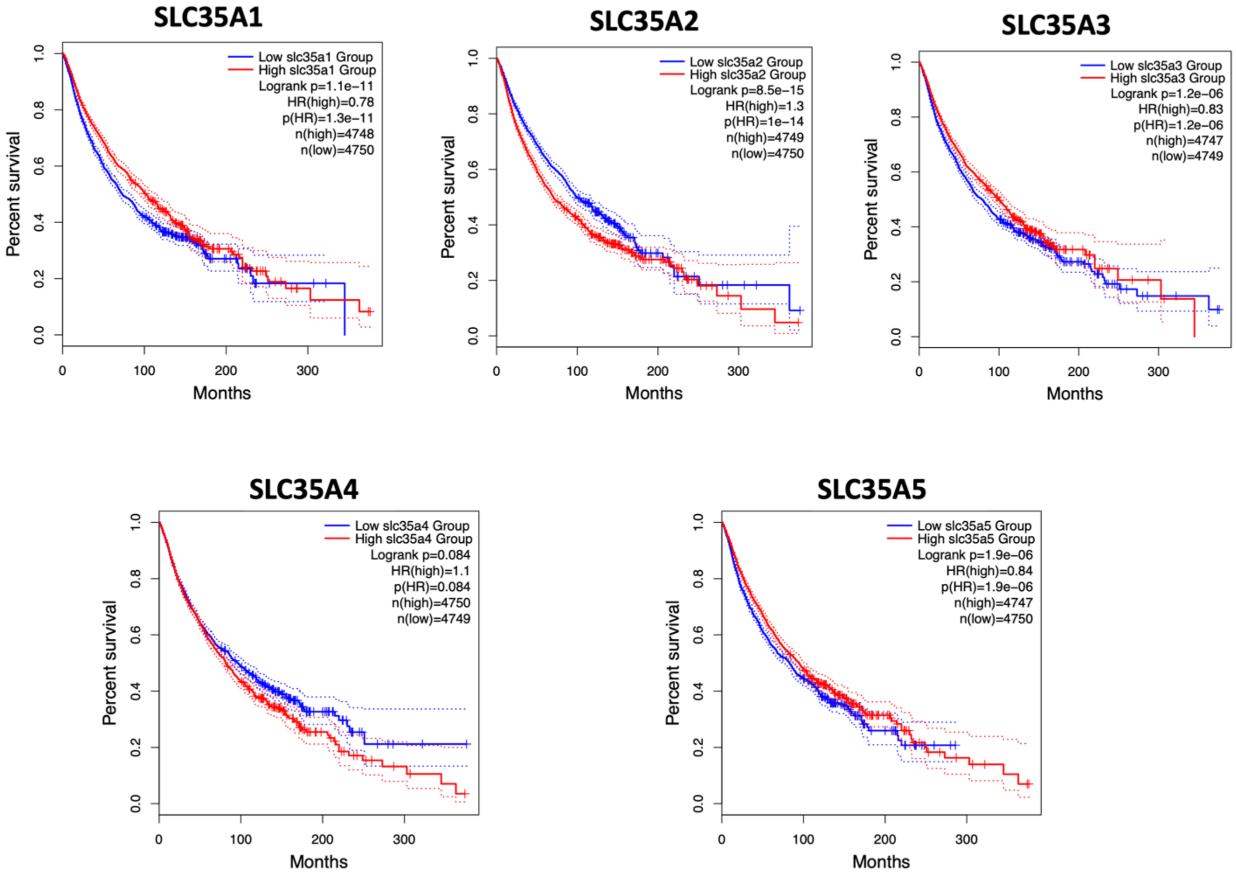

Figure S3. Recurrence metastasis-free survival (RFS) analysis of solute carrier 35A (SLC35A) family genes across several types of cancer (Kaplan-Meier plot). Red and blue curves represent survival analysis for higher and lower SLC35A mRNA expres-sion levels, respectively. All data were retrieved from TCGA Pan-Cancer Atlas, and p<0.05 was considered significant..

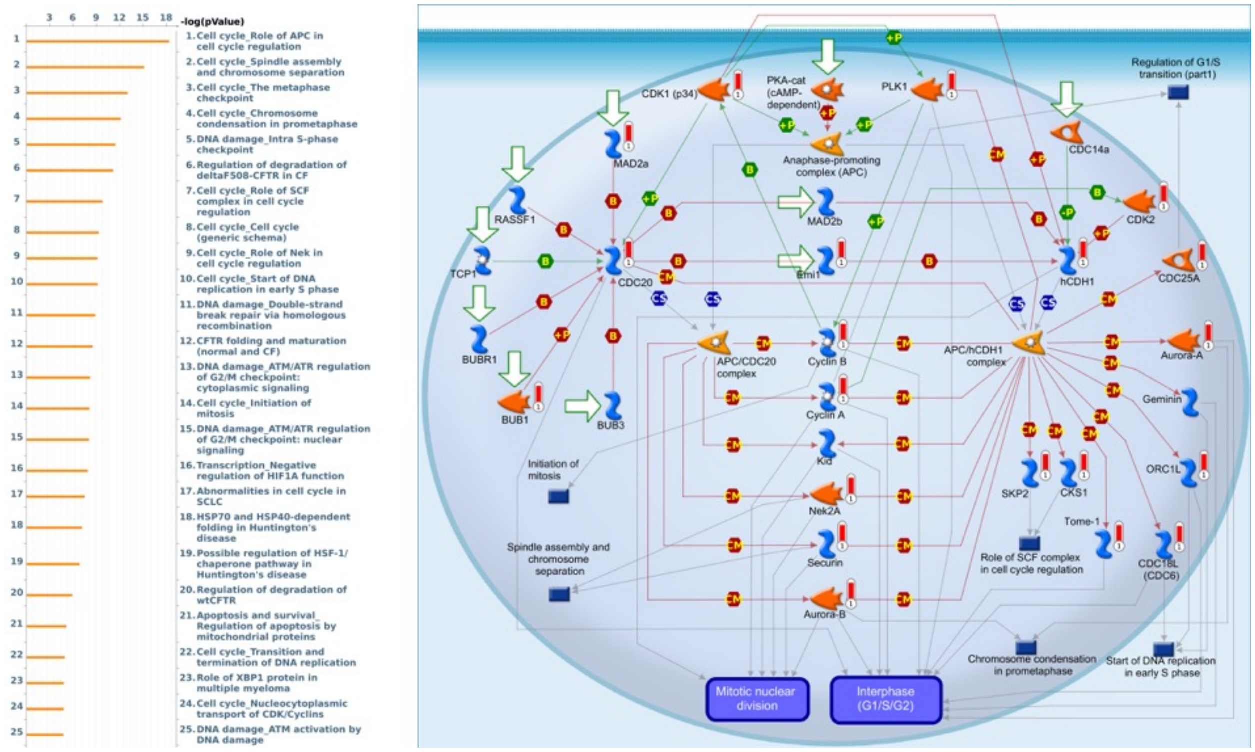

Figure S4. The approach was to collect the top 10% of expression gene lists from both METABRIC (2000 genes) and Pan-Cancer (1800 genes), and then overlap them to produce a final list of 1201 genes in total. The pathway list was ordered by the -log p value from the gene lists extracted via TCGA Pan-Cancer Atlas and METABRIC breast cancer patient databases. "Cell cycle_Role of APC in cell cy-cle regulation" was at the top of the pathway list when performing the "biological process" analysis.

Table S3. Pathway analysis of solute carrier 35A2 (SLC35A2)-coexpressed genes from pub-lic breast cancer databases using the MetaCore database (with p<0.01 set as the cutoff value)

#	Maps	pValue	Network Objects from Active Data
1	Cell cycle_Role of APC in cell cycle regulation	4,479E-19	CDC18L (CDC6), CDH1, Tome-1, Cyclin A, Aurora-B, CDC25A, SKP2, Cyclin B, ORC1L, CKS1, Nek2A, BUB1, Emi1, Aurora-A, PLK1, CDC20, MAD2a, Securin, CDK1 (p34), CDK2
2	Cell cycle_Spindle assembly and chromosome separation	8,198E-16	Importin (karyopherin)-alpha, RCC1, KNSL1, Aurora-B, HEC, Tubulin alpha, Cyclin B, Separase, ZW10, Nek2A, TPX2, CSE1L, Aurora-A, CDC20, MAD2a, Securin, CDK1 (p34), Tubulin (in microtubules)
3	Cell cycle_The metaphase checkpoint	1,209E-13	INCENP, SPBC25, Aurora-B, HEC, HZwint-1, ZW10, Survivin, CENP-E, Nek2A, BUB1, CENP-A, Aurora-A,

			PLK1, CDCA1, CDC20, CENP-F, MAD2a
4	Cell cycle_Chromosome condensation in prometaphase	9,585E-13	INCENP, Cyclin A, CNAP1, CAP-D2/D3, Aurora-B, Cyclin B, TOP2, Histone H3, CAP-G, CAP-G/G2, Aurora-A, Histone H1, CDK1 (p34)
5	DNA damage_Intra S-phase checkpoint	4,637E-12	CDC18L (CDC6), CDH1, Histone H2AX, Chk2, MCM3, Cyclin A, Chk1, CDC25A, MCM7, MCM2, Histone H3, BLM, FANCD2, DTL (hCdt2), MCM4, FANCI (KIAA1794), PP1-cat, HUWE1, MCM10, PP1-cat alpha, CDK2, CDC45L
6	Regulation of degradation of deltaF508-CFTR in CF	9,432E-12	HSP105, UFD1, VCP, Hdj-2, HSP90, Csp, CHIP, Sti1, HSP70, Aha1, SAE1, E2I, SUMO-2, Derlin1, SUMO-3, HSPBP1
7	Cell cycle_Role of SCF complex in cell cycle regulation	2,234E-10	Skp2/TrCP/FBXW, Chk1, CDC25A, SKP2, CKS1, CDK4, Emi1, Cyclin E, PLK1, NEDD8, CDK1 (p34), CDC34, CDK2
8	Cell cycle_Cell cycle (generic schema)	6,805E-10	Cyclin A, CDC25A, Cyclin B, E2F2, CDC25C, CDK4, E2F5, Cyclin E, CDC25B, CDK1 (p34), CDK2
9	Cell cycle_Role of Nek in cell cycle regulation	1,000E-09	Tubulin beta, Tubulin gamma, RCC1, HEC, Tubulin alpha, Histone H3, Nek2A, TPX2, Aurora-A, MAD2a, Histone H1, CDK1 (p34), Tubulin (in microtubules)
10	Cell cycle_Start of DNA replication in early S phase	1,000E-09	CDC18L (CDC6), MCM3, ORC6L, MCM4/6/7 complex, MCM2, ORC1L, RPA3, MCM4, Cyclin E, MCM10, Histone H1, CDK2, CDC45L
11	DNA damage_Double-strand break repair via homologous recombination	2,005E-09	RecQL4, DSS1, Histone H2AX, RMI2, EXO1, AUNIP, BLM, Rad51, RAD54B, RAD54L, PIR51, PLK1, Histone H2A, Histone H2B, MCM8, Histone H4, HROB, BRG1, CDK1 (p34), CDK2
12	CFTR folding and maturation (normal and CF)	4,217E-09	HSP40, HSP105, DNAJB6 (Hdj-1), Hdj-2, Csp, ERp29, Sti1, HSP70, Aha1, HSP90 beta, HSPBP1

13	DNA damage_ATM/ATR regulation of G2/M checkpoint: cytoplasmic signaling	9,461E-09	JAB1, BORA, Chk2, Chk1, Aurora-B, CDC25A, Histone H3, CDC25C, UBE2C, Aurora-A, PLK1, PP1-cat, CDC25B, CDK1 (p34), 14-3-3
14	Cell cycle_Initiation of mitosis	1,195E-08	Lamin B, KNSL1, Cyclin B2, Histone H3, CDC25C, PLK1, CDC25B, FOXM1, Kinase MYT1, Histone H1, CDK1 (p34)
15	DNA damage_ATM/ATR regulation of G2/M checkpoint: nuclear signaling	1,320E-08	CDC18L (CDC6), CDH1, HSF1, Histone H2AX, Chk2, Cyclin A, Chk1, Cyclin B, Cyclin B2, TTK, CDC25C, PLK1, CDK1 (p34), CDK2
16	Transcription_Negative regulation of HIF1A function	1,887E-08	HSP40, MCM3, MCM7, ARD1, VCP, MCM2, PSMA7, HSP90, Calpain 1(mu), PRDX4, CHIP, HSP70, RUVBL2, Sirtuin7, HSP90 beta, LAMP2, Elongin C
17	Abnormalities in cell cycle in SCLC	4,684E-08	Cyclin A, Aurora-B, SKP2, E2F2, Histone H3, CKS1, CDK4, Cyclin E, CDK1 (p34), Cyclin E2, CDK2
18	HSP70 and HSP40-dependent folding in Huntington's disease	1,070E-07	HSP40, PSMD1, DNAJB6 (Hdj-1), Hdj-2, HSP90, CHIP, Sti1, HSP70, HSP90 beta, Cathepsin D
19	Possible regulation of HSF-1/ chaperone pathway in Huntington's disease	2,319E-07	HSP40, HSF1, HSP90, PLA2, HSP70, E2I, PLK1, SUMO-2, HSP90 beta
20	Regulation of degradation of wtCFTR	2,100E-06	HSP105, UFD1, VCP, HSP90, Csp, CHIP, Derlin1, HSPBP1
21	Apoptosis and survival_Regulation of apoptosis by mitochondrial proteins	1,167E-05	Cathepsin L, Cyclin A, VDAC 1, Bax, PP2C, IFI27, MPTP complex, VDAC 2, Aif, Calpain 1(mu), TIMM8A, Metaxin 1, Smac/Diablo, 14-3-3 zeta/delta, PP1-cat alpha, Cathepsin D, CDK2
22	Cell cycle_Transition and termination of DNA replication	2,013E-05	TOP2 alpha, Cyclin A, MCM2, TOP2, POLD reg (p50), FEN1, CDK1 (p34), CDK2
23	Role of XBP1 protein in multiple myeloma	2,618E-05	SERP1, DNAJB11, GRP78, PSMA7, PSMA5, PSMA6, ERP5
24	Cell cycle_Nucleocytoplasmic transport of CDK/Cyclins	2,642E-05	Importin (karyopherin)-alpha, Cyclin A, CDK4, Cyclin E, CDK1 (p34), CDK2
25	DNA damage_ATM activation by DNA damage	2,946E-05	RecQL4, Histone H2AX, CDK5, SKP2, Histone H3, HSP90, Calpain 1(mu), TTI1, NK31, HSP90 beta,

			Histone H1.2, Histone H2B, Histone H4
26	Cell cycle_ESR1 regulation of G1/S transition	3,035E-05	Cyclin A, Skp2/TrCP/FBXW, CDC25A, SKP2, CKS1, CDK4, Cyclin A2, Cyclin E, CDK2
27	DNA damage_ATM/ATR regulation of G1/S checkpoint	3,537E-05	Histone H2AX, Chk2, Cyclin A, p70 S6 kinases, Chk1, CDC25A, CDK4, Cyclin E, CDK2, RFWD3
28	Cell cycle_Role of 14-3-3 proteins in cell cycle regulation	5,293E-05	Chk2, Chk1, CDC25A, CDC25C, 14-3-3 zeta/delta, CDC25B, CDK1 (p34)
29	DNA damage_ATM-dependent double-strand break foci	6,385E-05	Histone H2AX, SET8, UFD1, VCP, Histone H3, E2I, Histone H2A, Histone H1.2, Histone H2B, Histone H4, BRG1, Mi-2 alpha
30	Cell cycle_Sister chromatid cohesion	7,295E-05	Cyclin B, Separase, Histone H3, DCC1, Securin, Histone H1, CDK1 (p34)
31	Transcription_Role of heterochromatin protein 1 (HP1) family in transcriptional silencing	9,470E-05	Mi-2, CDC25A, Histone H3, Cyclin A2, Cyclin E, E2I, Histone H4, CDK1 (p34), Mi-2 alpha
32	Reproduction_Progesterone-mediated oocyte maturation	9,470E-05	CDC25C, BUB1, c-Src, Aurora-A, PLK1, CDC20, CDC25B, Kinase MYT1, CDK1 (p34)
33	Apoptosis and survival_Granzyme A signaling	1,162E-04	PAR1, Histone H3, NDPK A, HSP70, hnRNP C, Histone H2B, Histone H4, Lamin B1, Histone H1
34	Development_Glucocorticoid receptor signaling	1,315E-04	NCOA1 (SRC1), HSP90, HSP70, NCOA2 (GRIP1/TIF2), FKBP4, C/EBPbeta, E2I
35	Putative pathways of activation of monoclonal protein secretion in multiple myeloma	1,315E-04	SSR-delta, SERP1, DNAJB11, SRP-alpha, GRP78, ARMET, ERP5
36	Transport_RAN regulation pathway	1,384E-04	Importin (karyopherin)-alpha, RCC1, RanGAP1, NTF2, E2I, RanBP1
37	Immune response_Antigen presentation by MHC class I: cross-presentation	2,710E-04	SEC22B, Cathepsin L, HSP105, IP-30, HYOU1, DAP12, VCP, VAMP8, HSP60, HSP90, CHIP, HSP70, Endoplasmin, Calreticulin
38	HCV-dependent transcription regulation leading to HCC	3,573E-04	Bax, SKP2, CDK4, HSP70, Cyclin E, CDK2
39	GTP-XTP metabolism	3,598E-04	KGUA, RRP41, RPA16, GMPS, RPA39, POLR2D, POLR2J, NDPK A, HPRT, POLR3K, RPB8, IMD1, PNPH

40	Cigarette smoke-mediated regulation of NRF2-antioxidant pathway in airway epithelial cells	3,608E-04	ME1, TALDO, G6PD, PRDX1, TXNRD1, SRX1, Pirin
41	Cell adhesion_Gap junctions	4,714E-04	Tubulin beta, E-cadherin, Actin cytoskeletal, Tubulin alpha, Actin, Tubulin (in microtubules)
42	DNA damage_Nucleotide excision repair	4,840E-04	Centrin-2, EZH2, UFD1, DNA polymerase kappa, VCP, Histone H3, DTL (hCdt2), Histone H2A, Histone H2B, NEDD8, Histone H4
43	Apoptosis and survival_DNA-damage-induced apoptosis	5,142E-04	Histone H2AX, Chk2, Chk1, BLM, FANCD2
44	Epigenetic alterations in ovarian cancer	5,319E-04	DNMT3B, E-cadherin, EZH2, Bax, Aurora-B, SKP2, Histone H3, CDK4, Aurora-A, CDC20, Claudin-4, SSTR1
45	Signal transduction_mTORC1 downstream signaling	5,363E-04	4E-BP1, p70 S6 kinases, GLUT1, HMGCS2, G6PD, SCD, MTHFD2, p70 S6 kinase2, MVK, ATG13
46	NETosis in SLE	5,589E-04	Histone H3, Histone H2, Histone H2A, Histone H1.2, Histone H4, CAMP, Histone H1
47	LRRK2 in neurons in Parkinson's disease	8,350E-04	Actin cytoskeletal, MARK2, HSP90, CHIP, ACTB, Tubulin (in microtubules), 14-3-3
48	LRRK2 in neuronal apoptosis in Parkinson's disease	9,777E-04	Thioredoxin, VDAC 1, MPTP complex, ANT, Caspase-3
49	Immune response_Antigen presentation by MHC class I, classical pathway	1,009E-03	PSMB5, TAP1 (PSF1), CHIP, HSP70, BCAP31, Endoplasmin, Calreticulin, Impas 1, PSMB2
50	Oxidative stress_Role of ASK1 under oxidative stress	1,009E-03	Thioredoxin, HPK38, UNRIP, PRDX1, c-Src, MT-TRX, 14-3-3 zeta/delta, Glutaredoxin, 14-3-3
